# Correlation of intestinal bacteria, fungi and dietary nutrient intake in NAFLD patients with spleen deficiency syndrome

**DOI:** 10.3389/fcimb.2025.1586212

**Published:** 2025-08-11

**Authors:** Guiru Lin, Wanyi Ou, Jianmei Yang, Dongliang Chen, Yuanfei Wang, Aiping Wu, Lilian Gao, Wan Qu, Chenli Lin, Yinji Liang

**Affiliations:** ^1^ School of Nursing, Jinan University, Guangzhou, China; ^2^ Department of Metabolic and Bariatric Surgery, The First Affiliated Hospital, Jinan University, Guangzhou, China; ^3^ Centre of Health Management, The First Affiliated Hospital, Jinan University, Guangzhou, China; ^4^ School of Medicine, Jinan University, Guangzhou, China; ^5^ Health Science Center, Jinan University, Guangzhou, China

**Keywords:** non-alcoholic fatty liver disease, spleen deficiency syndrome, intestinal bacteria, intestinal fungi, dietary nutrient intake

## Abstract

**Background:**

Spleen deficiency syndrome (SDS) is one of the primary Traditional Chinese Medicine (TCM) syndromes in Non-alcoholic fatty liver disease (NAFLD). Diet influences NAFLD and SDS through the intestinal microbiota. The current study aimed to investigate the interrelationships of intestinal bacteria, fungi and dietary nutrient intake in NAFLD patients with SDS.

**Methods:**

The NAFLD TCM Patient Reported Outcome (PRO) Scale was administered to evaluate the TCM clinical symptoms of NAFLD patients. The Spleen Deficiency PRO Scale and Food Frequency Questionnaire (FFQ) were employed to respectively diagnose spleen deficiency syndrome and assess dietary nutrient intake, energy-adjusted dietary inflammatory index (E-DII), and dietary diversity scores (DDS) in NAFLD patients. Subsequently, stool samples were collected for 16S rRNA gene and ITS2 region sequencing to analyze the interrelationships among target intestinal bacteria, fungi, and dietary nutrient intake.

**Results:**

The NAFLD TCM PRO Scale indicated that the average score for symptoms related to SDS in NAFLD patients was 4.13 ± 0.40. Compared with NAFLD patients without SDS, those with SDS had insufficient dietary nutrient intake of diet-derived antioxidants such as carotene and folic acid, stronger pro-inflammatory effects of food, and reduced dietary diversity (*P* < 0.05). Additionally, sufficient dietary diversity was identified as a protective factor against SDS in NAFLD (OR: 0.424; 95% CI: 0.309, 0.583; *P* < 0.001). 16S rRNA gene and ITS2 region sequencing results showed that *Collinsella* (LDA = 3.947, *P =* 0.046) and *Rhizopus* (LDA = 3.196, *P =* 0.01) were enriched in NAFLD patients with SDS, whereas *Intestinimonas* was markedly increased in NAFLD patients without SDS (LDA = 2.015, *P* = 0.02). Correlation analysis demonstrated that *Gemmiger* and *Rhizopus* were significantly positively correlated (*r* = 0.778, *P* < 0.001), as were *Candida* and *Segatella* (*r* = 0.569, *P* < 0.001). *Intestinimonas* was positively correlated with the intake of antioxidant and anti-inflammatory nutrients such as dietary fiber, vitamin C, and iron (0.2 < *r* < 0.5, *P* < 0.05), while niacin intake was negatively correlated with *Rhizopus* abundance (*r* = -0.39, *P* = 0.025).

**Conclusion:**

Symptoms related to SDS are common in patients with NAFLD. The independent and interactive effects of intestinal bacteria and fungi might have collectively influenced the immune function and inflammation levels in NAFLD patients with SDS. These processes were likely associated with the intake of antioxidant and anti-inflammatory nutrients, as well as niacin.

## 1 Introduction

Non-alcoholic fatty liver disease (NAFLD) is the most prevalent chronic liver disease worldwide, characterized by excessive hepatic fat accumulation, affecting approximately 38% of the global population (Wong et al., 2023). Approximately 29.6% of the Chinese population is affected by NAFLD (Fan et al., 2024). NAFLD is also associated with a variety of extrahepatic diseases, such as cardiovascular disease, chronic kidney disease, and multiple extrahepatic cancers (Miao et al., 2024a), leading to substantial healthcare expenditures, economic losses, and decreased health-related quality of life (Lazarus et al., 2022). Traditional Chinese medicine (TCM), as a widely recognized form of complementary and alternative therapy, provides novel insights for the prevention and treatment of NAFLD, owing to its distinctive therapeutic approach (Zhang et al., 2024b). Our preclinical study confirmed that the TCM formula Shenling Baizhu San, known for its spleen-tonifying effects, significantly ameliorated NAFLD (Chen et al., 2024). Spleen deficiency syndrome (SDS) is one of the primary TCM syndromes in NAFLD (Dai et al., 2022). NAFLD patients with SDS often present with symptoms such as decreased appetite, loose stool, insomnia, and increased susceptibility to infections (Dai et al., 2022; Zheng et al., 2020). Therefore, early identification of symptoms and biomarkers in NAFLD patients with SDS may effectively reduce the economic burden and improve the quality of life for these patients.

Intestinal microbiota influences the progression of both NAFLD and SDS. Comprising intestinal bacteria, fungi, and viruses, intestinal microbiota exerts critical functions in maintaining intestinal homeostasis, immune responses, and microbial balance through bacterial-fungal interactions (Li et al., 2024a). Intestinal microbiota dysbiosis increases intestinal permeability, potentially exposing the liver to harmful substances, thereby promoting hepatic fat accumulation and liver fibrosis (Hu et al., 2020). Current evidence indicates that the pathogenesis of SDS is closely related to intestinal microbiota dysbiosis, with significant intestinal bacterial disturbances observed in both SDS mouse models and patients (Peng et al., 2014, 2020; Yu et al., 2024). Dietary nutrient intake directly modulates intestinal microbiota composition, which manifests through increased intestinal permeability and elevated inflammatory mediators, thereby accelerating liver disease progression (Jiménez-González et al., 2025). Understanding the impact of diet on the interaction between the intestinal microbiota and the host immune system may provide valuable insights for developing nutritional strategies to maintain intestinal health in NAFLD patients with SDS (Zhang, 2022).

Currently, most research focuses on the relationship between NAFLD and intestinal microbiota, while the role of intestinal microbiota in NAFLD patients with SDS remains unclear, particularly regarding the role of intestinal fungi. Therefore, we collected stool samples from NAFLD patients with and without SDS and performed 16S rRNA gene and ITS2 region sequencing to investigate the characteristics of intestinal bacteria and fungi. Additionally, we explored the interactions between dietary nutrient intake and intestinal microbiota in NAFLD patients with SDS, ultimately providing novel insights into the prevention, treatment, and dietary management of NAFLD patients with SDS.

## 2 Materials and methods

### 2.1 Diagnostic criteria

The diagnosis of NAFLD was established according to the Asia-Pacific Working Party on NAFLD guidelines, incorporating three essential criteria: 1) B-ultrasound examination confirmed hepatic steatosis; 2) absence or minimal alcohol consumption (defined as < 70 g/week for women and < 140 g/week for men); and (3) exclusion of alternative etiologies of hepatic steatosis, including but not limited to autoimmune liver diseases and viral hepatitis (Wong et al., 2018). The diagnostic criterion for SDS was based on the Spleen Deficiency PRO scale developed by the Institute of Longhua Hospital, Shanghai University of Traditional Chinese Medicine (SR1362577) (Dai et al., 2022). Patients with a total score ≥ 20 were identified as NAFLD patients with spleen deficiency syndrome (NAFLD_P), whereas those with a score < 20 were categorized as NAFLD patients without spleen deficiency syndrome (NAFLD_NP).

### 2.2 Patient recruitment

This study included patient from patients visiting the First Affiliated Hospital of Jinan University from January to October 2024. Detailed inclusion criteria: 1) Meet the diagnostic criterion; 2) Voluntary participation in the study with signed informed consent; 3) Age range 18 to 70 years; 4) According to the requirements of the FFQ, participants had resided in Guangzhou for at least 6 months and had an energy intake within the normal range. The general energy intake range is 500–3500 kcal/day for females and 800–4000 kcal/day for males; 5) Participants from whom stool samples were collected must not have used antibiotics, probiotics, prebiotics, hormones, immunosuppressants, or Chinese herbs in the past 3 months (Miao et al., 2024b). Participants with any of the following conditions were excluded from the study: Individuals with severe systemic or infectious diseases, such as malignancies, severe cardiopulmonary disorders, neurological diseases, HIV infection, etc.

This study was approved by the Medical Ethics Review Board of Jinan University (Approval No. JUNKY-2023-0130) and was supervised by the Ethics Committee throughout the research process. Written informed consent was obtained from all participants prior to their enrollment in the study.

### 2.3 Research tools

#### 2.3.1 The NAFLD traditional Chinese medicine reported outcome scale (The NAFLD TCM PRO)

We assessed the TCM clinical symptoms in NAFLD patients using the NAFLD TCM PRO scale. This self-assessment tool enables participants to evaluate their condition based on subjective feelings. Comprising three domains and nine subdomains, the scale has undergone multiple validations and demonstrates robust reliability and validity, with a total Cronbach’s α coefficient exceeding 0.8. Utilizing a 5-point Likert rating system, higher scores on the scale indicate better health status and quality of life (Shen, 2017).

#### 2.3.2 The spleen deficiency reported outcome scale

Patients were asked to score the severity of ten symptoms based on their own experience, and the final symptom score was calculated by multiplying the individual symptom scores by their corresponding weights. This questionnaire has been validated for reliability among the study population, demonstrating good reliability and validity with a Cronbach’s α coefficient of 0.773, which indicates acceptable internal consistency (Dai et al., 2022).

#### 2.3.3 The food frequency questionnaire

The FFQ used in this study encompasses the range of foods commonly consumed by individuals in the Guangzhou area. The survey examines the participants’ dietary habits over the past six months, with a focus on three primary aspects: food types, frequency of consumption, and the average intake. The questionnaire includes 7 major food categories and 82 individual food items. Dietary data were collected through one-on-one, face-to-face interviews during patient clinic visits, and food portion reference charts were provided to aid participants in accurately estimating their portion sizes (Zhang and Ho, 2009).

### 2.4 Research methods

#### 2.4.1 Dietary nutrient intake

Dietary nutrient intake was computed from the FFQ data. Nutrient intake was energy-adjusted using the nutrient density method, whereby participants’ original daily dietary nutrient intakes were converted to intake amounts per 1000 kcal (Mccullough and Byrd, 2023).

#### 2.4.2 Energy-adjusted dietary inflammatory index

The calculation of the Dietary Inflammatory Index (DII) was based on the dietary data collected through the FFQ. For each food item, actual intake values were standardized using the global dietary standard library’s average intake and standard deviation. Z-scores were computed and then converted into percentile values. These values were subsequently doubled and subtracted by 1, resulting in a zero-centered, symmetrical distribution. The DII score for each food item was obtained by multiplying the percentile value by the corresponding inflammatory effect score. The total DII score was calculated by summing the individual DII scores of all food items. E-DII employed in this study followed a similar calculation method, but prior to Z-score conversion, the raw dietary nutrient intake values were adjusted to correspond to a 1000 kcal energy intake. Similarly, the average intake and standard deviation in the global dietary standard library were also adjusted for a 1000 kcal energy intake (Shivappa et al., 2014; Chen et al., 2022).

#### 2.4.3 Dietary diversity scores

The food items collected through the FFQ were categorized into nine groups based on the Chinese Dietary Guidelines (2022): cereal, vegetables, fruits, Soybeans and their products, eggs, meat, fish, milk and dairy products, and oil. The frequency of food intake was classified into five levels: “almost every day”, “not every day, but once a week at least”, “not every week, but once a month at least”, “not every month, but sometimes”, “seldom or never”. In this study, participants were assigned 1 point for consuming a food item “almost every day” or “once a week at least”, and 0 points for all other frequencies. Each food category was scored only once. A higher score reflects a higher dietary diversity level, with the maximum possible score being 9 (Zhu et al., 2024).

### 2.5 Stool sample collection

The researcher prepared disposable sterile 5 ml stool collection tubes (Manufacturer: Beijing BIORIDA Technology Co., Ltd., Catalog No. BA-0206) in advance and instructed the patients on stool collection procedures. Approximately 2–3 g of stool sample for each patient was collected from the inside (not the surface) using clean stool collection paper and a sterile spoon to avoid contamination by urine or water. After collection, the sample must be immediately placed in a portable ice box and promptly transferred to a -80°C refrigerator for storage until further processing (Kyriazopoulou et al., 2025).

### 2.6 Gut microbiota detection

HiPure Stool DNA was extracted using the HiPure Stool DNA Extraction Kit (Magen, Guangzhou, China). PCR amplification was conducted for the V3-V4 hypervariable region of the 16S rRNA gene and the ITS2 region (16S rRNA primer sequences: 5’-3’, 341F: CCTACGGGNGGCWGCAG, 806R: GGACTACHVGGGTATCTAAT; ITS2 primer sequence: 5’-3’, ITS3_KYO2: GATGAAGAACGYAGYRAA, ITS4: TCCTCCGCTTATTGATATGC). Subsequently, Illumina sequencing was performed, and 2% agarose gel electrophoresis was used to preliminarily assess the quality of the amplification products. The PCR products were purified using AMPure XP Beads (Beckman, CA, USA). The purified products were quantified using Qubit 3.0, and sequencing libraries were constructed using the Illumina DNA Prep Kit (Illumina, CA, USA). The quality of the libraries was assessed using the ABI StepOnePlus Real-Time PCR System (Life Technologies, Foster City, USA). Qualified libraries were sequenced and analyzed on the NovaSeq 6000 platform using the PE250 mode. All quality control and sequencing procedures were conducted by GENEDENOVO Biotechnology Company (Guangzhou, China). The obtained data were quality-controlled, clustered, and de-chimerized to obtain operational taxonomic units (OTUs). Species annotation was performed based on OTU sequences to obtain species abundance information at each level. Subsequently, bioinformatics analysis were performed on species composition (Krona, version 2.6), indicator species (R VennDiagram package, version 1.6.16), Alpha diversity (QIIME, version 1.9.1), Beta diversity (Muscle, version 3.8.31), functional prediction (PICRUSt, version 2.1.4), and environmental factor association (R Vegan Package, version 2.5.3) (Caporaso et al., 2010; Chen and Boutros, 2011).

### 2.7 Statistical methods

Continuous variables that followed a normal distribution were expressed as mean ± standard deviation. When the assumption of equal variances was met, group comparisons were performed using Student’s t-test; otherwise, Welch’s t-test was used. Non-normally distributed variables were expressed as medians (interquartile range), with group comparisons conducted using the Mann-Whitney U test. Categorical variables were presented as counts (percentages) and analyzed using the chi-square test. Pearson, mantel test, and procrustes analysis were employed to assess the relationships between variables. Binary logistic regression was used to identify the influencing factors of NAFLD patients with SDS. A P-value of < 0.05 was considered statistically significant.

## 3 Results

### 3.1 The NAFLD TCM PRO scale scores

In this study, a total of 388 NAFLD patients completed the NAFLD TCM PRO scale. The general information of the patients and the laboratory examination results were detailed in the **Supplementary Material** (**Supplementary Table S1-2**). The scores of NAFLD patients in various domains were presented in **Table 1-1**, with the lowest average item score observed in the treatment domain (3.75 ± 0.66) points. The detailed scores for each aspect of the physiological domain in NAFLD patients were presented in **Table 1-2**, with the total score for symptoms related to SDS being (20.70 ± 2.14) points, a mean item score of (4.13 ± 0.40) points, ranking third. **Table 1–3** specifically displayed the symptoms related to SDS item scores, among which the item “Do you experience loose or unformed stools?” showed the lowest mean score of (3.82 ± 0.87) points.

Table 1-1Scoring in various domains of the NAFLD TCM PRO scale.

**Table:** 

Domain	Total score( $\bar{x}$ ± *s)*	Mean item score( $\bar{x}$ ± *s)*
Physiological Domain	108.95 ± 9.95	4.19 ± 0.38
Psychological Domain	17.66 ± 2.28	4.41 ± 0.57
Treatment Domain	11.24 ± 1.97	3.75 ± 0.66

**Table 1–2:** Scoring of physiological domain items in the NAFLD TCM PRO scale.

Physiological domain	Total score ( $\bar{x}$ ± *s)*	Mean item score ( $\bar{x}$ ± *s)*
Symptoms related to qi deficiency syndrome	8.24 ± 1.45	4.12 ± 0.73
Symptoms related to yin deficiency syndrome	16.75 ± 2.75	4.19 ± 0.69
Symptoms related to spleen deficiency syndrome	20.70 ± 2.14	4.13 ± 0.40
Symptoms related to phlegm dampness trapped spleen syndrome	16.60 ± 2.57	4.15 ± 0.64
Symptoms related to liver and spleen damp-heat syndrome	23.99 ± 2.83	4.00 ± 0.47
Symptoms related to liver qi stagnation syndrome	13.74 ± 1.41	4.58 ± 0.47
Symptoms related to liver stomach disharmony syndrome	8.97 ± 1.20	4.48 ± 0.60

**Table 1–3:** Scoring of symptoms related to spleen deficiency syndrome items in the NAFLD TCM PRO scale.

Symptoms related to spleen deficiency syndrome	Mean item score ( $\bar{x}$ ± *s)*
How has your appetite been since the onset of your illness?	4.31 ± 0.74
Have you experienced a decrease in appetite?	4.38 ± 0.66
Has your complexion worsened, appearing dull, sallow, or pale?	4.06 ± 0.70
Do you experience a bland or tasteless sensation in your mouth?	4.08 ± 0.75
Do you experience loose or unformed stools?	3.82 ± 0.87

### 3.2 Participant characteristics

#### 3.2.1 Baseline characteristics

In total, 245 NAFLD patients were recruited, comprising 106 cases of NAFLD_NP (43.27%) and 139 cases of NAFLD_P (56.73%). The spleen deficiency score in NAFLD_P patients was significantly higher than that in NAFLD_NP patients (12.03 ± 4.67 *vs*. 35.31 ± 11.59, *P* < 0.001). Compared to NAFLD_NP patients, NAFLD_P patients exhibited significant differences in personal income (*P* = 0.018), weekly exercise time (*P* = 0.002), daily sleep duration (*P* = 0.03), and family history of liver disease (*P* = 0.037), as detailed in **Table 2-1**. Laboratory examination results revealed that total protein (TP, *P* = 0.009) and albumin (ALB, *P* = 0.035) levels in NAFLD_P were lower than in NAFLD_NP, while total cholesterol (TC, *P* = 0.008) and low-density lipoprotein (LDL, *P* = 0.039) levels were significantly high in NAFLD_P compared to NAFLD_NP, with further details provided in **Table 2-2**.

Table 2-1Baseline characteristics of NAFLD_NP and NAFLD_P patients.

**Table:** 

Variable	NAFLD_NP (n=106)	NAFLD_P (n=139)	*t/χ* ^2^ */Z*	*P*
Gender				
Male	75 (70.8%)	90 (64.7%)	0.987	0.321
Female	31 (29.2%)	49 (35.3%)		
Age (years)	34 (27,40)	33 (28,39)	-0.357	0.721
Height (cm)	169 (162,173)	168.5 (162,174)	-0.202	0.840
Weight (kg)	85 (77,100)	87.9 (76.4,106.3)	-0.564	0.573
BMI (kg/m²)	29.8 (27.4,34.2)	30.5 (27.1,37.3)	-0.542	0.588
Marital status				
Unmarried	44 (41.5%)	50 (36.0%)	2.350	0.552
Married	59 (55.7%)	81 (58.3%)		
Divorced	2 (1.9%)	7 (5.0%)		
Widowed	1 (0.9%)	1 (0.7%)		
Personal Monthly Income (CNY/month)				
0	13 (12.3%)	6 (4.3%)	10.124	**0.018**
<0, <5000	8 (7.5%)	25 (18.0%)		
5000-10000	43 (40.6%)	59 (42.4%)		
>10000	42 (39.6%)	49 (35.3%)		
Educational level				
Junior High School and Below	13 (12.3%)	12 (8.6%)	2.649	0.266
High School and Junior College	23 (21.7%)	42 (30.2%)		
Bachelor’s Degree and Above	70 (66.0%)	85 (61.2%)		
Occupation				
Employed	82 (77.4%)	120 (86.3%)	3.590	0.309
Student	5 (4.7%)	4 (2.9%)		
Retired	11 (10.4%)	10 (7.2%)		
Other	8 (7.5%)	5 (3.6%)		
Weekly Moderate-Intensity Exercise Time (minutes/week)				
0	51 (48.1%)	95 (68.3%)	11.989	**0.002**
>0, <150	33 (31.1%)	32 (23.1%)		
150	22 (20.8%)	12 (8.6%)		
Daily sleep duration (hours/night)				
<6	15 (14.2%)	35 (25.2%)	5.915	**0.030**
6-8	91 (85.8%)	102 (73.4%)		
>8	0 (0%)	2 (1.4%)		
Smoking				
Yes	22 (20.8%)	28 (20.1%)	0.014	0.906
No	84 (79.2%)	111 (79.9%)		
Alcoholism				
Yes	19 (17.9%)	33 (23.7%)	1.217	0.270
No	87 (82.1%)	106 (76.3%)		
Diabetes				
Yes	30 (28.3%)	34 (24.5%)	0.460	0.498
No	76 (71.7%)	105 (75.5%)		
Hypertension				
Yes	18 (17.0%)	32 (23.0%)	1.351	0.245
No	88 (83.0%)	107 (77.0%)		
Family History of Liver Disease				
Yes	2 (1.9%)	11 (7.9%)	4.348	**0.037**
No	104 (98.1%)	128 (92.1%)		
Duration of NAFLD ≥5 Years				
Yes	8 (7.5%)	17 (12.2%)	1.439	0.230
No	98 (92.5%)	122 (87.8%)		
Spleen Deficiency Score	12.03 ± 4.67	35.31 ± 11.59	-19.507	**<0.001**

Values in bold indicate statistically significant differences (*P* < 0.05).

**Table 2–2:** Laboratory data of NAFLD_NP and NAFLD_P patients.

Variable	NAFLD_NP (n=106)	NAFLD_P (n=139)	*t/Z*	*P*
Low-Density Lipoprotein (mmol/L)	3.21 ± 0.65	3.40 ± 0.75	-2.081	**0.039**
Total Protein (g/L)	73.61 ± 5.24	71.70 ± 6.02	2.651	**0.009**
Albumin (g/L)	45.13 ± 3.19	44.14 ± 3.88	2.124	**0.035**
Globulin (g/L)	28.88 ± 3.92	28.49 ± 4.18	0.755	0.451
Creatinine (umol/L)	73.60 ± 17.33	70.01 ± 18.34	1.554	0.122
High-Density Lipoprotein (mmol/L)	1.20 ± 0.25	1.23 ± 0.27	-0.980	0.328
Total Cholesterol (mmol/L)	5.3 (4.6,6)	5.6 (5,6.1)	-2.663	**0.008**
White Blood Cell Count (×10^9^/L)	7.7 (6.4,8.9)	7.6 (6.8,8.9)	-0.694	0.488
Triglycerides (mmol/L)	1.7 (1.2,2.5)	1.7 (1.2,2.7)	-0.066	0.948
Alanine Aminotransferase (U/L)	37 (23,59)	38 (22,61)	-0.354	0.723
Aspartate Aminotransferase (U/L)	26 (19,36)	26 (19,39)	-0.533	0.594
Gamma-Glutamyl Transferase (U/L)	37 (24,56)	45 (26,66)	-1.767	0.077
Albumin/Globulin Ratio	1.6 (1.4,1.7)	1.5 (1.4,1.7)	-0.819	0.413
Glucose (mmol/L)	5.3 (4.7,6.9)	5.3 (4.9,6.6)	-0.392	0.695

Values in bold indicate statistically significant differences (*P* < 0.05).

**Table 2–3:** Dietary nutrient intake of NAFLD_NP and NAFLD_P patients.

Variable	NAFLD_NP (n=106)	NAFLD_P (n=139)	*t/χ* ^2^ */Z*	*P*
Protein (g)	40.09 ± 9.29	39.54 ± 8.37	0.486	0.628
Total fat (g)	49.23 ± 7.68	49.04 ± 7.57	0.190	0.849
Carbohydrate (g)	101.69 ± 18.65	103.01 ± 17.12	-0.576	0.565
Vitamin E (mg)	15.21 ± 3.86	14.87 ± 3.72	0.710	0.478
Niacin (mg)	11.22 ± 2.56	10.98 ± 2.52	0.738	0.461
Manganese (mg)	2.67 ± 0.61	2.53 ± 0.61	1.836	0.068
Phosphorus (mg)	560.44 ± 111.64	545.48 ± 93.96	1.138	0.256
Monounsaturated Fatty Acids (g)	18.10 ± 3.10	17.93 ± 3.18	0.442	0.659
Polyunsaturated Fatty Acids (g)	12.36 ± 2.85	12.04 ± 2.84	0.857	0.392
Dietary fiber (g)	4.08 (3.37,5.35)	3.96 (3.24,4.81)	-1.288	0.198
Cholesterol (mg)	305.13 (200.02,387.63)	270.52 (192.63,366.24)	-1.346	0.178
Vitamin A (ug RE)	492.47 (382.42,656.83)	423.77 (301.81,586.63)	-2.613	**0.009**
Carotene (ug)	1937.28 (1367.78,2591.07)	1600.46 (1117.58,2499.72)	-2.485	**0.013**
Vitamin B1 (mg)	0.53 (0.44,0.63)	0.53 (0.44,0.63)	-0.158	0.874
Vitamin B2 (mg)	0.65 (0.52,0.77)	0.60 (0.50,0.75)	-1.421	0.155
Vitamin C (mg)	57.48 (38.68,73.64)	50.36 (35.60,70.69)	-1.823	0.068
Calcium (mg)	285.60 (228.02,388.94)	292.08 (218.16,374.37)	-0.378	0.705
Potassium (mg)	982.83 (803.34,1138.23)	938.83 (806.28,1094.80)	-1.161	0.246
Sodium (mg)	480.69 (368.78,578.09)	501.52 (390.85,599.13)	-1.123	0.262
Magnesium (mg)	141.20 (125.97,164.27)	142.93 (121.17,160.29)	-0.817	0.414
Iron (mg)	11.33 (10.26,12.57)	10.93 (9.70,12.25)	-2.112	**0.035**
Iodine (ug)	15.52 (10.80,19.51)	13.69 (10.19,19.31)	-1.445	0.149
Zinc (mg)	7.25 (6.23,8.40)	7.00 (6.18,8.20)	-0.890	0.374
Selenium (ug)	34.65 (27.80,41.56)	34.12 (28.15,44.05)	-0.713	0.476
Copper (mg)	2.60 (1.12,7.20)	3.91 (1.51,8.99)	-1.896	0.058
Saturated Fatty Acids (g)	12.28 (10.60,13.69)	12.46 (10.80,14.31)	-0.517	0.605
Choline (mg)	29.03 (22.72,36.41)	27.14 (21.99,34.03)	-1.341	0.180
Betaine (mg)	113.08 (63.79,185.44)	84.06 (53.01,130.07)	-2.360	**0.018**
Folic acid (ug)	166.35 (145.18,196.88)	157.17 (131.64,189.68)	-2.262	**0.024**
Vitamin B6 (mg)	0.57 (0.51,0.66)	0.57 (0.47,0.64)	-1.250	0.211
Vitamin B12 (ug)	1.83 (1.41,2.42)	1.83 (1.33,2.33)	-0.566	0.571

Values in bold indicate statistically significant differences (*P* < 0.05).

**Table 2–4:** The E-Dll score of NAFLD_NP and NAFLD_P patients.

Variable	NAFLD_NP (n=106)	NAFLD_P (n=139)	*t*	*P*
E-Dll	0.02 ± 1.20	0.35 ± 1.18	-2.205	**0.028**

Values in bold indicate statistically significant differences (*P* < 0.05).

**Table 2–5:** The dietary diversity score of NAFLD_NP and NAFLD_P patients.

Variable	NAFLD_NP (n=106)	NAFLD_P (n=139)	*χ* ^2^/*t*	*P*
Cereal				
No	0 (0%)	0 (0%)	-	-
Yes	106 (100%)	139 (100%)		
Soybeans and their products				
No	34 (32.1%)	86 (61.9%)	21.364	**<0.001**
Yes	72 (67.9%)	53 (38.1%)		
Vegetables				
No	0 (0%)	0 (0%)	-	**-**
Yes	106 (100%)	139 (100%)		
Fruits				
No	7 (6.6%)	31 (22.3%)	11.309	**0.001**
Yes	99 (93.4%)	108 (77.7%)		
Meat				
No	0 (0%)	0 (0%)	-	-
Yes	106 (100%)	139 (100%)		
Fish				
No	39 (36.8%)	71 (51.1%)	4.962	**0.026**
Yes	67 (63.2%)	68 (48.9%)		
Eggs				
No	4 (3.8%)	13 (9.4%)	2.899	0.089
Yes	102 (96.2%)	126 (90.6%)		
Milk and dairy products				
No	35 (33.0%)	65 (46.8%)	4.702	**0.030**
Yes	71 (67.0%)	74 (53.2%)		
Oil				
No	0 (0%)	0 (0%)	-	-
Yes	106 (100%)	139 (100%)		
Dietary Diversity Score	7.88 ± 0.91	7.09 ± 1.08	6.068	**<0.001**

Values in bold indicate statistically significant differences (*P* < 0.05).

**Table 2–6:** Logistic regression analysis of factors influencing spleen deficiency in NAFLD patients.

Variable	OR (95%Cl)	*P*
Vitamin A	0.999 (0.998,1.001)	0.233
Carotenoids	0.999 (0.999,1.000)	0.054
Iron	0.993 (0.785,1.257)	0.954
Betaine	1.000 (0.995,1.004)	0.858
Folic acid	0.998 (0.989,1.007)	0.706
E-Dll	0.547 (0.286,1.046)	0.068
DDS	0.424 (0.309,0.583)	**<0.001**

#### 3.2.2 Dietary nutrient intake

The comparative analysis of dietary nutrient intake (per 1000 kcal), as shown in **Table 2-3**, revealed that NAFLD_P patients had insufficient dietary nutrient intake of vitamin A (*P* = 0.009), carotenoids (*P* = 0.013), iron (*P* = 0.035), betaine (*P* = 0.018), and folate compared (*P* = 0.024) to NAFLD_NP patients. Although food had a pro-inflammatory effect in both NAFLD_NP and NAFLD_P patients, the E-Dll score of NAFLD_P patients was higher than that of NAFLD_NP patients, indicating that food had a stronger pro-inflammatory effect in NAFLD_P patients (0.02 ± 1.20 *vs*. 0.35 ± 1.18, *P* = 0.028, **Table 2-4**). The dietary diversity score of NAFLD_P was lower than that of NAFLD_NP (7.88 ± 0.91 *vs*. 7.09 ± 1.08, *P* < 0.001, **Table 2-5**). Notably, the number of NAFLD_NP patients who consumed soy and soy products (*P* < 0.001), fruits (*P* = 0.001), fish (*P* = 0.026), milk (*P* = 0.03), and dairy products on a weekly basis was markedly higher than that of NAFLD_P patients.

#### 3.2.3 Logistic regression analysis of factors influencing spleen deficiency in NAFLD patients

Indicators with statistically significant differences in dietary analysis between NAFLD_NP and NAFLD_P patients were included in logistic regression analysis. The results (**Table 2-6**) indicated that only sufficient dietary diversity is a protective factor against spleen deficiency in NAFLD patients (OR:0.424; 95% CI:0.309, 0.583; *P* < 0.001). A one-unit increase in dietary diversity score was associated with a 0.426-fold decrease in the risk of spleen deficiency in NAFLD patients.

### 3.3 Characteristics of intestinal microbiota

#### 3.3.1 Characteristics of intestinal bacteria

**Figure 1:**
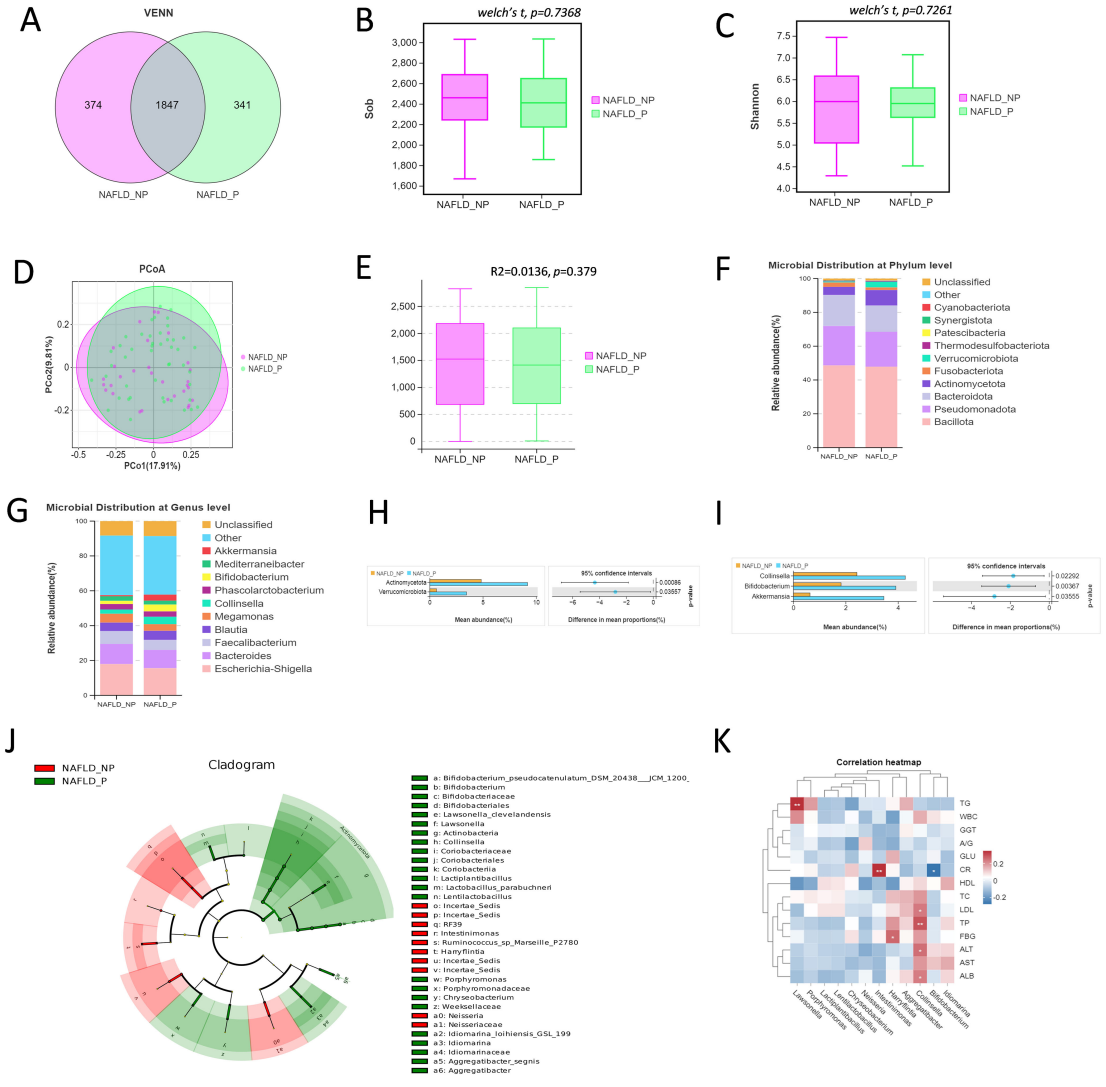
Intestinal bacteria in the NAFLD_NP and NAFLD_P patients. **(A)** Comparison of intestinal bacteria OTUs between NAFLD_NP and NAFLD_P patients. **(B, C)** Alpha diversity is indicated by Sob index and Shannon diversity index. **(D)** Principal coordinate analysis (PCoA) based on bray. **(E)** Adonis analysis of similarities, R2 = 0.0136, *P* = 0.379. **(F)** Top 10 most relatively abundant intestinal bacteria at the phylum level. **(G)** Top 10 most relatively abundant intestinal bacteria at the genus level. **(H)** Significant differences in intestinal bacteria at the phylum level (top 10). **(I)** Significant differences in intestinal bacteria at the genus level (top 10). **(J)** Cladogram of 57 differential intestinal bacteria with LEfSe analysis (LDA > 2, *P* < 0.05). **(K)** Pearson correlation analysis of the relative abundance of differential potential biomarker intestinal bacteria at the genus level with biochemical indices. *P < 0.05 or **P < 0.01.

To further investigate the potential role of intestinal bacteria in NAFLD with SDS, a total of 76 stool samples (30 for NAFLD_P, 46 for NAFLD_NP). The sequencing results revealed that there were 1,847 OTUs common to both groups, with 341 OTUs unique to the NAFLD_P patients and 374 OTUs unique to the NAFLD_NP patients (**Figure 1A**). The alpha diversity analysis showed that there was no statistically significant differences in both the Sob and Shannon index between the NAFLD_P and NAFLD_NP patients (*P* = 0.737 for Sob index, *P* = 0.727 for Shannon index, Welch’s t-test, **Figures 1B, C**). Beta diversity was evaluated using Bray-Curtis distances via principal component analysis (PCoA) combined with the Adonis (PERMANOVA) test, indicating no significant structural differences between the two groups (*P* = 0.379, **Figures 1D, E**). The relative abundance of species in the top 10 at the phylum and genus levels for the two groups is shown in **Figures 1F, G**, respectively. The relative abundances of *Actinomycetota* (*P* < 0.001) and *Verrucomicrobiota* (*P* = 0.036) at the phylum level, and *Akkermansia* (*P* = 0.036), *Bifidobacterium* (*P* = 0.004), and *Collinsella* (*P* = 0.023) at the genus level, were higher in NAFLD_P patients than in NAFLD_NP patients (**Figures 1H, I**).

LEfSE analysis was used to determine the contribution of intestinal bacteria in distinguishing between NAFLD_P and NAFLD_NP. As shown in **Figure 1J**, the results revealed that 18 intestinal bacteria were significantly enriched in the NAFLD_P group, while 7 intestinal bacteria were enriched in the NAFLD_NP group with LDA > 2 and *P* < 0.05. It is noteworthy that the relative abundance of *Collinsella* is significantly high in the NAFLD_P patients (LDA=3.947, *P* = 0.046), while *Intestinimonas* is significantly high in the NAFLD_NP patients (LDA= 2.015, *P* = 0.02). Further association of differential intestinal bacteria at the genus level with biochemical indices revealed that *Collinsella* was positively correlated with Alanine Aminotransferase (ALT, *r =* 0.262, *P =* 0.022), LDL (*r =* 0.246, *P =* 0.032), TP (*r =* 0.302, *P =* 0.008), and ALB (*r =* 0.24, *P =* 0.037), while *Intestinimonas* is positively correlated with CR (*r* = 0.353, *P* = 0.002, **Figure 1K**).

#### 3.3.2 Characteristics of intestinal fungi

**Figure 2:**
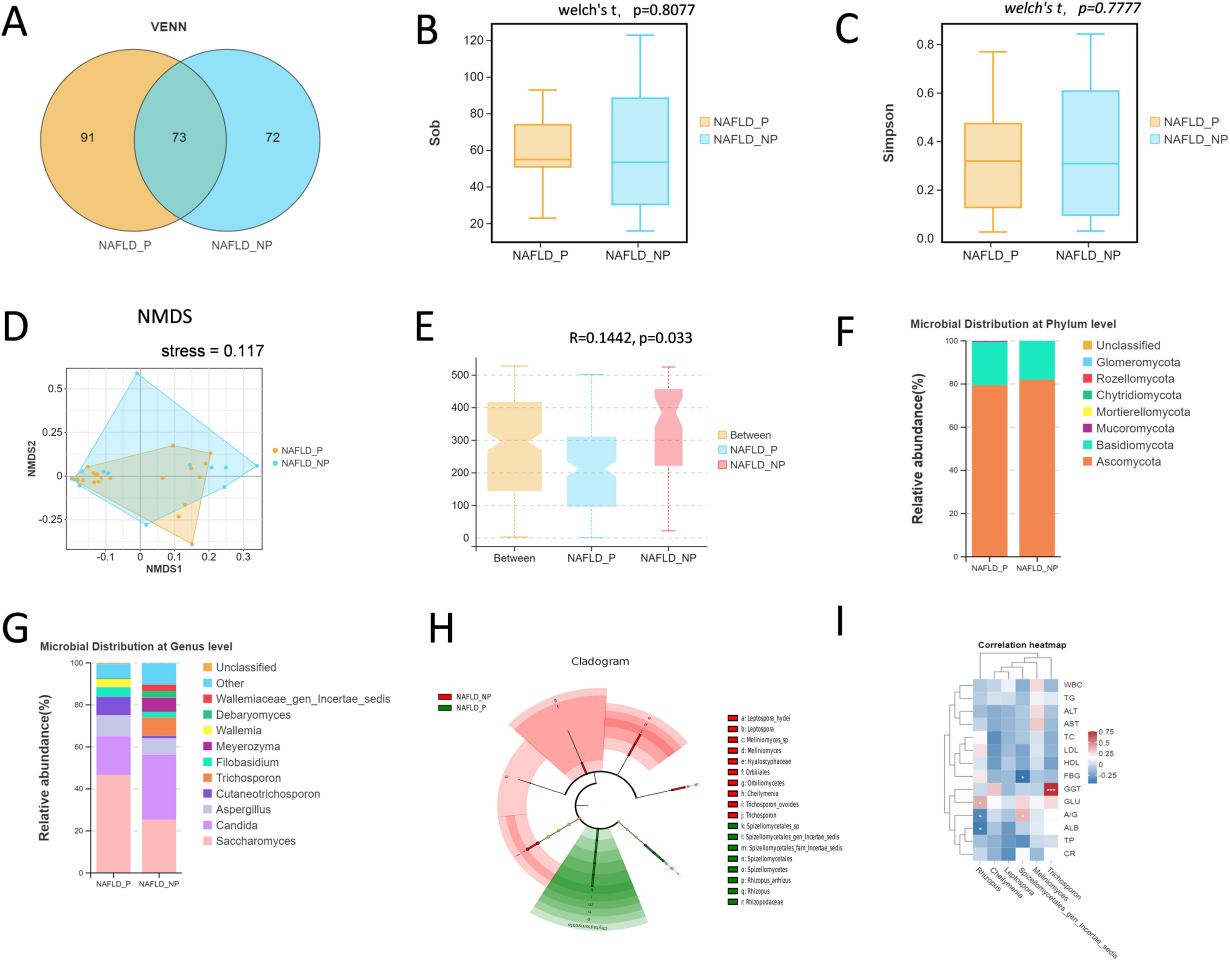
Intestinal fungi in the NAFLD_NP and NAFLD_P patients. **(A)** Comparison of intestinal fungi OTUs between NAFLD_NP and NAFLD_P patients. **(B, C)** Alpha diversity is indicated by Sob index and Simpson diversity index. **(D)** Non-metric multidimensional scaling (NMDS) based on bray. **(E)** NMDS Analysis of similarities (ANOSIM), If 1 > R > 0, it suggests that the inter-group differences exceed the intra-group variations, R = 0.1442, *P* = 0.033. **(F)** Top 10 intestinal fungi at phylum level. **(G)** Top 10 intestinal fungi at the genus level. **(H)** Cladogram of 19 differential intestinal fungi with LEfSe analysis (LDA > 2, *P* < 0.05). **(I)** Pearson correlation analysis of the relative abundance of differential potential biomarker intestinal fungi at the genus level with biochemical indices. *P < 0.05 or ***P < 0.001.

33 stool samples were subjected to ITS2 region sequencing analysis to characterize the intestinal fungi in NAFLD patients (21 for NAFLD_P, 12 for NAFLD_NP). A total of 73 OTUs were shared between the two groups, with 72 OTUs unique to the NAFLD_NP group and 91 OTUs unique to the NAFLD_P group (**Figure 2A**). The Shannon and Simpson indices were used to reflect the abundance and evenness of the intestinal fungi. The results showed that there was no significant differences between the two groups (*P* = 0.808 for Sob index, *P* = 0.778 for Simpson index, Welch’s t-test, **Figures 2B, C**). Beta diversity was analyzed via Non-metric Multidimensional Scaling (NMDS) and Analysis of Similarities (ANOSIM). Although the NMDS plot showed some overlap between groups (**Figure 2D**), significant differences in fungal composition were observed, with inter-group differences being greater than intra-group differences (R = 0.1442, *P* = 0.033, **Figure 2E**). The composition of the top ten intestinal fungi at both the phylum and genus levels in the NAFLD_NP and NAFLD_P patients was shown in **Figures 2F ,G**. The result of LEfSe analysis indicated that 9 and 10 intestinal fungi were respectively enriched in NAFLD_P and NAFLD_NP patients with LDA > 2, *P* <0.05 (**Figure 2H**). Notably, the *Rhizopus* was significantly enriched in the NAFLD_P patients and is classified as an opportunistic pathogen capable of causing mucormycosis (LDA = 3.196, *P =* 0.01) (Petrikkos et al., 2012). Further correlation analysis between differential intestinal fungi at the genus level and biochemical indices revealed that Rhizopus was negatively correlated with ALB (*r* = -0.392, *P* = 0.024, **Figure 2I**).

#### 3.3.3 Correlation analysis between intestinal bacteria and fungi

**Figure 3:**
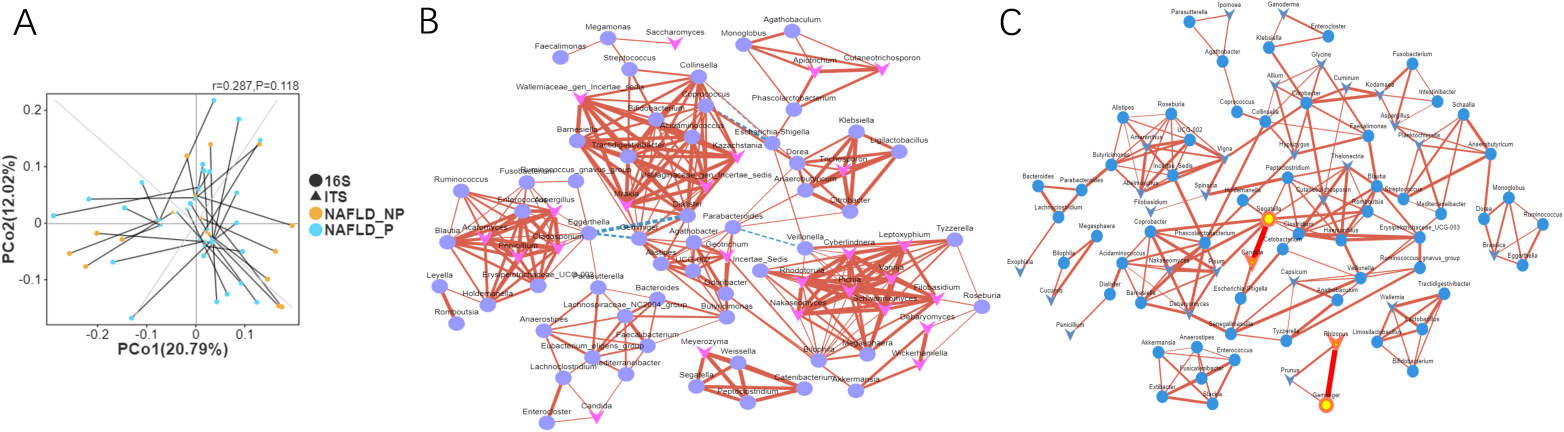
The association between intestinal bacteria and fungi in NAFLD_NP and NAFLD_P patients. **(A)** Procrustes analysis of intestinal bacteria and fungal community structures between NAFLD_P and NAFLD_NP patients based on Bray-Curtis distance at the OTU level. **(B)** Pearson correlation network of bacteria and fungi at the genus level in the NAFLD_NP patients. **(C)** Pearson correlation network of bacteria and fungi at the genus level in the NAFLD_P patients. The networks are composed of nodes and edges, where nodes represent species and edges represent correlations, circles are used to denote bacteria, and triangles denote fungi, the size of the nodes indicates the degree of connectivity, solid red lines represent positive correlations, while dashed blue lines represent negative correlations. *P* value: 0 to 0.05, correlation index: -1 to -0.5 or 0.5 to 1.

In the Procrustes analysis based on Bray distance at the OTU level, we found that the community structures of intestinal bacteria and fungi did not differ significantly between NAFLD_P and NAFLD_NP patients. Additionally, samples were widely dispersed in the principal coordinate space, reflecting low similarity of community structures (M²= 0.917, *P* = 0.118, **Figure 3A**). Consequently, we conducted a network analysis of intestinal bacteria and fungi at the genus level in both NAFLD_P and NAFLD_NP patient groups and identified several potentially meaningful associations. The results demonstrated that bacteria-fungi associations were stronger in NAFLD_NP patients compared to NAFLD_P patients(**Figures 3B, C**). Nevertheless, meaningful associations were still observed in NAFLD_P patients, including a strong positive correlation between intestinal bacteria *Gemmiger* and fungi *Rhizopus* (*r* = 0.778, *P* < 0.001), as well as between intestinal bacteria *Segatella* and fungi *Candida* (*r* = 0.569, *P* < 0.001).

### 3.4 Correlation analysis between the intestinal microbiota and dietary nutrient intake

**Figure 4:**
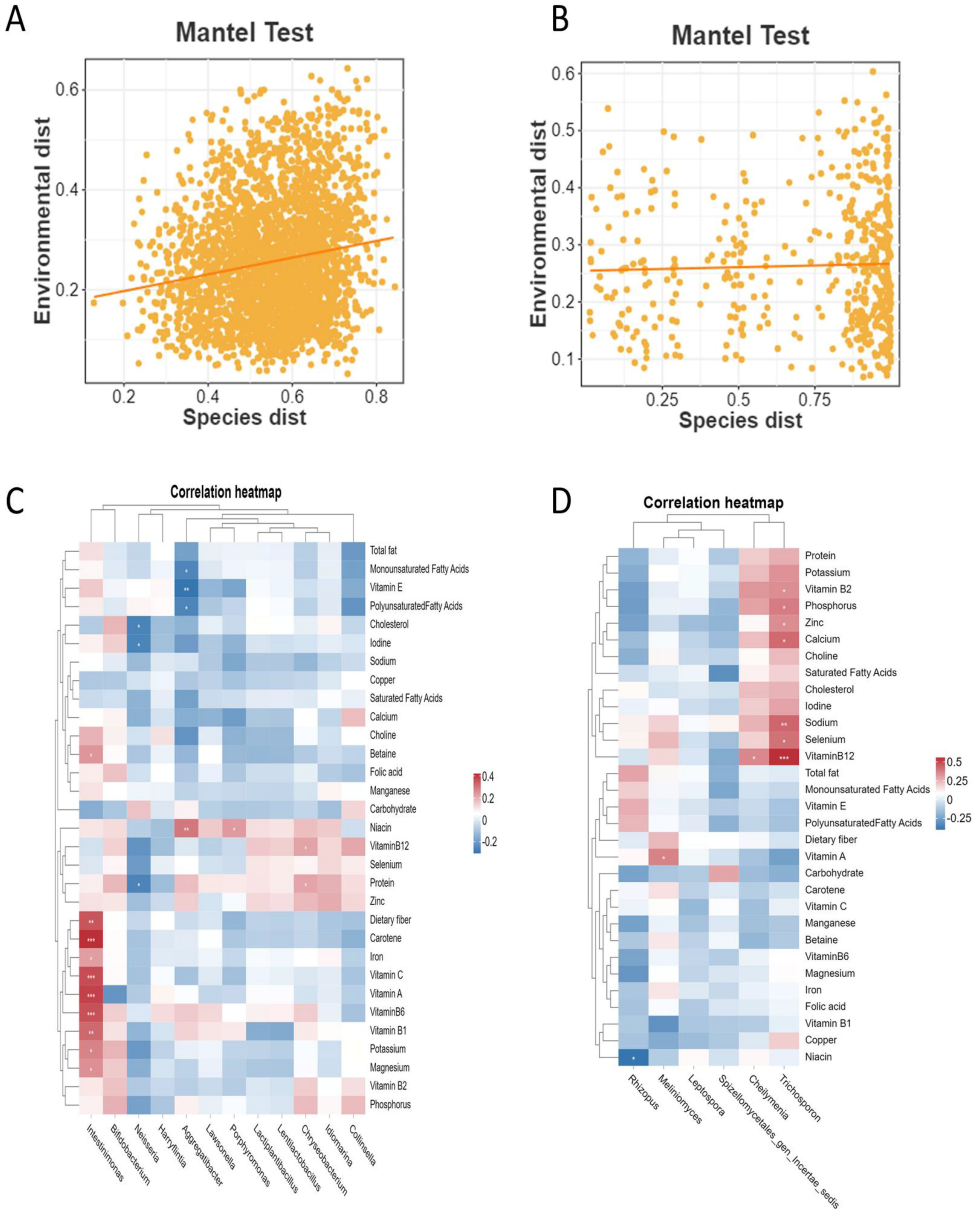
The association analysis of dietary nutrient intake and intestinal microbes in NAFLD_NP and NAFLD_P patients. **(A)** Mantel test result for the correlation between the OTU distance matrix of intestinal bacteria and dietary nutrient intake distance matrix. **(B)** Mantel test result for the correlation between the OTU distance matrix of intestinal fungi and dietary nutrient intake distance matrix. **(C)** A heatmap illustrating the correlation between the relative abundance of differential potential intestinal bacteria and dietary nutrient intake. **(D)** A heatmap illustrating the correlation between the relative abundance of differential potential intestinal fungi and dietary nutrient intake. Pearson correlation analysis was conducted to assess the association between differential potential biomarker intestinal microbes at the genus level and dietary nutrient intake in both the NAFLD_NP and NAFLD_P patients. *P < 0.05, **P < 0.01, ***P < 0.001.

The Mantel test results showed a significant weak correlation between the OTU distance matrix of intestinal bacteria and dietary nutrient intake distance matrix (*r* = 0.167, *P* < 0.001, **Figure 4A**), while no significant association was found between the OTU distance matrix of intestinal fungi and dietary nutrient intake distance matrix (*r* = 0.044, *P* = 0.259, **Figure 4B**). Pearson correlation analysis was further employed to explore the relationship between the relative abundances of genus-level differential potential biomarkers in the intestinal microbiota and dietary nutrient intakes. The results revealed significant positive correlations between the relative abundance of *Intestinimonas* and dietary fiber, carbohydrate (*r =* 0.364, *P <* 0.001), vitamin A (*r =* 0.397, *P <* 0.001), vitamin B1 (*r = 0*.331, *P =* 0.004), vitamin B6 (*r =* 0.371, *P <* 0.001), vitamin C (*r =* 0.376, *P <* 0.001), iron (*r =* 0.236, *P =* 0.04), potassium (*r =* 0.284, *P =* 0.013), and magnesium (*r =* 0.262, *P =* 0.022) (**Figure 4C**). Additionally, a significant negative correlation was found between the relative abundance of *Rhizopus* and Niacin (*r* = -0.39, *P* = 0.025, **Figure 4D**). Furthermore, the relative abundance of intestinal fungi *Trichosporon* exhibited significant positive correlations with vitamin B2 (*r =* 0.351, *P =* 0.045), vitamin B12 (*r =* 0.567, *P < 0.001*), sodium (*r =* 0.444, *P =* 0.01), and calcium (*r =* 0.437, *P =* 0.011).

## 4 Discussion

Patient self-management played a crucial role in the progression of NAFLD (Kwon et al., 2023). This study revealed that NAFLD patients scored lowest in the treatment domain, reflecting poorer therapeutic adherence, which might result from insufficient disease knowledge, complex treatment protocols, and inadequate self-management strategies. Notably, we also found that symptoms associated with SDS were relatively common among NAFLD patients, with loose or unformed stools being the most frequent symptom. Typical symptoms and signs could effectively reflect the TCM syndromes of patients. The weakened spleen’s impaired transport function and the deficient stomach’s inability to digest led to food-fluid retention, which disrupts spleen yang ascent and ultimately caused diarrhea (Fan et al., 2023). Consequently, SDS might play a critical role in the occurrence and development of NAFLD, and this study will further explored the mechanism of SDS in NAFLD.

Engaging in at least 150–240 minutes of moderate aerobic exercise per week or sleeping 6–8 hours daily can effectively reduced hepatic steatosis in patients (Keating et al., 2023; Mikolasevic et al., 2021). In this study, fewer NAFLD patients with SDS engaged in 6–8 hours of daily sleep duration and 150 minutes of moderate-intensity exercise per week compared to those without SDS. Additionally, lack of physical activity weakened spleen transport and transformation functions, led to insufficient qi and blood production, which in turn affected sleep quality due to impaired nourishment of the heart and mind (Chen, 2019). Spleen deficiency might also lead to metabolic disorders such as lipid metabolism, amino acid metabolism, and energy metabolism (Li et al., 2023; Sun et al., 2024; Zhan et al., 2024), as evidenced by our results showed that NAFLD patients with SDS exhibited abnormalities in TP, ALB, LDL, and total cholesterol (TG) levels. In accordance with TCM, a deficiency in the spleen and stomach’s ability to transform food into essential nutrients resulted in inadequate production of qi and blood, caused a deficiency in the five zang-fu organs, diminished resistance to pathogenic factors, immune system imbalances, and ultimately inflammation (Qiu et al., 2024). Diet-derived antioxidants served as ideal supplements for NAFLD prevention, effectively inhibiting inflammation. In accordance with TCM, the insufficiency of spleen and stomach to convert food into essential nutrients might weaken the body’s resistance to disease factors, disrupt the immune system, and ultimately lead to inflammation (Chen et al., 2023). In the current study, evaluation of nutrient intake and the E-DII scores revealed significantly low daily levels of vitamin A, carotene, iron, betaine, and folic acid in NAFLD patients with SDS compared to those without. Carotene, iron, betaine, and folic acid were confirmed to have the function of inhibiting hepatic lipid accumulation and ameliorating hepatic oxidative stress (Hu et al., 2024; Liu et al., 2024b; Ma et al., 2025; Tyczynska et al., 2024). Despite the acknowledged antioxidant properties of vitamin A, the association between dietary vitamin A intake and the risk of NAFLD remained inconclusive (Liu et al., 2024a). Current research suggested that supplementation with specific vitamins and minerals might alleviate NAFLD symptoms, but the optimal dosage range remained unclear (Tyczynska et al., 2024). Sufficient dietary diversity was a protective factor for NAFLD patients with SDS. We also assessed the DDS of NAFLD patients, finding that patients with SDS had lower DDS scores than those without. Sufficient dietary diversity offered a richer selection of foods, and increased the intake of beneficial substances such as micronutrients and dietary fiber, thereby reducing NAFLD risk (Luo et al., 2022).

In the NAFLD population, *Collinsella* is notably high in patients with liver depression and spleen deficiency compared to those with damp-heat depression syndrome (Ma, 2022). Besides, *Collinsella*, as a pro-inflammatory genus, is also significantly enriched in NASH (Astbury et al., 2020). Previous research showed that Administration of jujube with spleen-tonifying properties to spleen deficiency rats for ten days significantly improved the relative abundance of *Collinsella* (Li, 2017). Current results showed that *Collinsella* is significantly enriched in NAFLD patients with SDS. *Collinsella* has been reported to influence host lipid metabolism via specific pathways (Astbury et al., 2020), and our findings further demonstrated that there was significant positive correlation between *Collinsella* and LDL, ALT, as well as TP levels. This correlation suggests that *Collinsella* may be implicated in metabolic dysregulation by modulating both lipid metabolic and protein synthetic processes. Additionally, study have indicated that, in a spleen deficiency rat model, the abundance of *Collinsella* and *Intestinimonas* was significantly increased and decreased, respectively (Sun, 2020). Furthermore, Butyrate, a common short-chain fatty acid, played a crucial role in reducing inflammation, maintaining colon health, and supporting the intestinal barrier (Cai et al., 2020). As a beneficial bacterium with anti-inflammatory and anti-obesity properties (Cai et al., 2020), accumulating evidences indicated that *Intestinimonas* was capable of fermenting fiber to produce butyrate and converting fructoselysine, an advanced glycation end-product, into butyrate (Niu et al., 2024; Kahleova et al., 2023). Similarly, *Intestinimonas* is significantly enriched in NAFLD patients without SDS in our study. Further analysis also revealed a positive correlation between the relative abundance of *Intestinimonas* and Cr, and low Cr levels has been reported as a predictor of NASH (Wu et al., 2021). It is speculated that NAFLD patients without SDS have a low risk of progressing to hepatitis compared to NAFLD patients with SDS.

Concurrently, previous studies indicated that Akkermansia and Bifidobacterium, recognized as beneficial bacteria, may increase in response to enhanced dietary carbohydrate bioavailability and can rise compensatory under disease conditions to restore microbial balance (Cox et al., 2021; Fava et al., 2013; Hizo and Rampelotto, 2023). Notably, excessive increases in microbial abundance may compromise gut microbiota stability and diversity, leading to metabolic dysfunction. Akkermansia, a mucin-degrading bacterium, may impair the intestinal barrier and promote LPS translocation when overabundant, thereby triggering low-grade inflammation (Qu et al., 2023). In our results, the significant increase in Akkermansia and Bifidobacterium relative abundance in NAFLD_P patients may be attributed to the factors mentioned above. Future studies should further investigate specific roles of these bacterium in NAFLD patients with SDS and evaluate the potential as therapeutic targets or biomarkers.

*Rhizopus* is a distinguishing fungi in metabolic-associated fatty liver disease, differentiating it from alcohol-related liver disease (Zhang et al., 2024a). Mucor sp. (a common opportunistic pathogen) and *Rhizopus* belong to the same order, Mucorales, and Mucor sp. has been shown to be positively correlated with liver inflammation and fibrosis (Demir et al., 2022). Besides, activation of innate immunity, as the first line of defense, plays a crucial role in liver inflammation in NAFLD (Arrese et al., 2016). Prior study showed that *Rhizopus* was the most common genus responsible for fatal mucormycosis infections in immunocompromised patients (Petrikkos et al., 2012). Correspondingly, a significant enrichment of Rhizopus also existed in NAFLD patients with SDS. Moreover, ALB has anti-inflammatory and antioxidant functions (Yang et al., 2021), while NAFLD patients exhibited lower ALB levels due to hepatic lipid accumulation, which might exacerbate the progression of liver inflammation (Peiseler et al., 2022; Li et al., 2024b). In our study, the relative abundance of *Rhizopus* was negatively correlated with ALB. Compared to NAFLD patients without SDS, those with SDS might have even low levels of ALB due to the pro-inflammatory effects of *Rhizopus*. Therefore, we hypothesize that the enrichment of pro-inflammatory bacteria and fungi in NAFLD patients with SDS is associated with elevated inflammation levels and impaired immunity.

Intestinal bacteria and fungi exhibit complex interrelationships in health and disease. In this study, though no significant association was detected between the community structures of intestinal bacteria and fungi, we identified potential meaningful interactions in NAFLD patients with SDS. In these patients, *Candida* was positively correlated with *Segatella*. Previous studies have shown that fungi could directly or indirectly reduce the levels of *Sutterella* (Scanu et al., 2024). *Sutterella*, a pathogenic intestinal microorganism linked to intestinal inflammation and gastrointestinal disorders, degrades IgA to impair intestinal immune function, thereby facilitating the invasion of pathogenic commensal bacteria (Singh et al., 2024; Kaakoush, 2020). Additionally, previous study demonstrated that the relative abundance of *Candida* significantly increased in mice fed high-fat and high-fructose diets, and was positively correlated with the NAFLD activity score (Zheng et al., 2024). *Candida albicans*, a common *Candida* species frequently found in NAFLD patients, was not only able to activate host Toll-like receptors(TLR), leading to the release of pro-inflammatory cytokines (Li et al., 2024a; Netea et al., 2006, 2002), but also to induce epigenetic reprogramming of innate immune cells (Netea et al., 2015). In addition, we also observed a positive correlation between *Rhizopus* and *Gemmiger*. In NAFLD patients with SDS and compromised immune function, *Rhizopus* promoted inflammation, while *Gemmiger* induced inflammation and related symptoms by downregulating TLR1 expression (Zhou et al., 2023). The limited biodiversity of bacteria and fungi in these patients may drive novel cross-kingdom interactions, potentially affecting host immune and inflammatory regulation (Yadav et al., 2022; Sokol et al., 2017). Therefore, we hypothesize that the interactions between *Rhizopus* and *Gemmiger*, as well as between *Candida* and *Segatella*, in NAFLD patients with SDS may collectively modulate the host’s immune and inflammatory responses.

Intestinal bacteria are not only associated with long-term diet but also influenced by factors such as exercise, pharmacologic factors, and host genetic factors (Chang and Kao, 2019; Zhang et al., 2025; Hoffmann et al., 2013). In the present study, long-term dietary intake was characterized using the food frequency questionnaire, and the results revealed a weak correlation between the OTU distance matrix of intestinal bacteria and dietary nutrient intake distance matrix. This suggests that, in addition to dietary influences, other complex factors may be involved in regulating the changes in intestinal bacteria in NAFLD patients with SDS. However, dietary intervention could still serve as a potential regulatory strategy. In contrast, no significant association was observed between the OTU distance matrix of intestinal fungi and dietary nutrient intake distance matrix, which may be attributed to the low abundance of fungi in the gut and their more rapid response to short-term dietary changes (Hoffmann et al., 2013). It is worth noting that *Intestinimonas* is an anti-inflammatory beneficial bacterium that ferments fiber to produce butyrate (Kahleova et al., 2023). In this study, we found that the relative abundance of *Intestinimonas* was found to be positively correlated not only with dietary fiber intake but also with carbohydrates, vitamins A, B1, B6, and C intake, as well as with iron potassium, and magnesium intake. Consequently, NAFLD patients without SDS showed *Intestinimonas* enrichment, potentially associated with high intake of antioxidant and anti-inflammatory foods compared to SDS patients. Furthermore, niacin intake was negatively correlated with the relative abundance of *Rhizo*pus. Niacin intake in NAFLD patients was lower than in healthy controls, particularly among those with SDS. Although the relationship between niacin and NAFLD is not fully understood, niacin could exert beneficial effects by reducing triglyceride synthesis and improving lipid profiles, and to mitigated free radical damage and reduce inflammation, which was crucial to alleviate NAFLD (Antentas et al., 2024; Zhou and Han, 2024). In our study, the relative abundance of *Rhizopus* was negatively correlated with the albumin-to-globulin (A/G) ratio, which was a potential biomarker of liver damage to some extent. Therefore, we speculate that dietary niacin may contribute to liver tissue damage through its interaction with *Rhizopus.* In the future, the optimal intake of niacin will still require further validation studies.

There are two main limitations in this study. First, the observational design without intervention precluded causal inference between intestinal microbiota and NAFLD patients with SDS. Second, the absence of metabolomic analyses prevented comprehensive characterization of microbiota-metabolome interactions. In future, studies should integrate longitudinal interventions with multi-omics approaches.

## 5 Conclusions

In this study, symptoms related to SDS were more common in NAFLD patients. The intake of dietary nutrients in NAFLD patients with SDS might have exhibited a pro-inflammatory effect, potentially due to insufficient intake of antioxidant nutrients. The independent and interactive effects of intestinal bacteria and fungi might have collectively influenced the immune function and inflammation levels in NAFLD patients with SDS. These processes were likely associated with the intake of antioxidant and anti-inflammatory nutrients, as well as niacin. These findings provide a theoretical basis for the clinical treatment and dietary management of NAFLD patients with SDS.

## Data Availability

The raw gut microbiome sequencing data from this study have been deposited in the NCBI Sequence Read Archive (SRA) with BioProject accession number PRJNA1299057.
