# Supplementary material for: Correlation of intestinal bacteria, fungi and dietary nutrient intake in NAFLD patients with spleen deficiency syndrome
Source: Front Cell Infect Microbiol. 2025 Aug 11;15:1586212. doi: 10.3389/fcimb.2025.1586212 (PMC12375650; doi:10.3389/fcimb.2025.1586212)
Supplement: Supplementary file 1 [file Table1.pdf]

Supplementary table 1-1 Baseline characteristics of NAFLD patients

Variables	Category	N (%)
Gender		
	Male	276(74.7%)
	Female	112(25.3%)
Age(years)		
	≤19	9(2.4%)
	20-39	279(71.9%)
	40-59	89(22.9%)
	60-79	11(2.8%)
BMI(kg/m^2)		
	< 23	12(3.1%)
	≥23	376(96.9%)
Marital status		
	Unmarried	159(41.0%)
	Married	215(55.4%)
	Divorced	12(3.1%)
	Widowed	2(0.5%)
Personal Monthly	0	26(6.7%)
	> 0, < 5000	61(15.7%)
	5000-10000	162(41.8%)
	> 10000	139(35.8%)
Educational level		
	Junior High School and Below	70(18.0%)
	High School and Junior College	161(41.5%)
	Bachelor's Degree and Above	157(40.5%)
Occupation		
	Employed	318(82.0%)
	Student	43(11.1%)
	Retired	13(3.3%)
	Other	14(3.6%)
Weekly Moderate-Intensity Exercise Time (minutes/week)		
	0	162(41.8%)
	> 0, < 150	158(40.7%)
	≥150	68(17.5%)

daily sleep duration (hours/night)		
	< 6	84(21.7%)
	6-8	298(76.8%)
	> 8	6(1.5%)
Smoking		
	Yes	81(20.9%)
	No	307(79.1%)
Alcoholism		
	Yes	76(19.6%)
	No	312(80.4%)
Diabetes		
	Yes	70(18.1%)
	No	318(81.9%)
Hypertension		
	Yes	57(14.7%)
	No	331(85.3%)
Family History of Liver Disease		
	Yes	17(4.4%)
	No	371(95.6%)
Duration of Fatty Liver ≥5 Years		
	Yes	54(13.9%)
	No	334(86.1%)

Supplementary table 1-2 Laboratory data of NAFLD patients

	Total Number	Elevated (N, %)	Normal (N, %)	Decreased (N, %)
White Blood Cell Count (×10^9/L)	374	64（17.11%）	310（82.89%）	0（0.00%）
Total Cholesterol (mmol/L)	352	132（37.50%）	218（61.93%）	2（0.57%）
Triglycerides	350	171（48.86%）	179（51.14%）	0（0.00%）

(mmol/L)				
High-Density Lipoprotein (mmol/L)	309	4（1.29%）	227（73.46%）	78（25.24%）
Low-Density Lipoprotein (mmol/L)	309	37（11.97%）	261（84.47%）	11（3.56%）
Alanine Aminotransferase (U/L)	383	187（48.83%）	196（51.17%）	0（0.00%）
Aspartate Aminotransferase (U/L)	383	106（27.68%）	270（70.50%）	7（1.82%）
Gamma-Glutamyl Transferase (U/L)	314	143（45.54%）	170（54.14%）	1（0.03%）
Total Protein (g/L)	251	8（3.19%）	229（91.24%）	14（5.58%）
Albumin (g/L)	251	1（0.40%）	226（90.04%）	24（9.56%）
Globulin (g/L)	251	3（1.20%）	246（98.01%）	2（0.80%）
Albumin/Globulin Ratio	251	0（0.00%）	233（92.83%）	18（7.17%）
Glucose (mmol/L)	364	1（0.27%）	264（72.53%）	99（27.20%）
Creatinine (umol/L)	375	210（56.00%）	158（42.13%）	7（1.87%）
